# Food safety labelling of chicken to prevent campylobacteriosis: consumer expectations and current practices

**DOI:** 10.1186/s12889-018-5322-z

**Published:** 2018-03-27

**Authors:** Philip D. Allan, Chloe Palmer, Fiona Chan, Rebecca Lyons, Olivia Nicholson, Mitchell Rose, Simon Hales, Michael G. Baker

**Affiliations:** Department of Public Health, University of Otago, PO Box 7343, Wellington, 6242 New Zealand

**Keywords:** Campylobacter, Food labelling, Food safety, Consumer expectations, Chicken, Poultry, Label

## Abstract

**Background:**

*Campylobacter* is the leading cause of bacterial gastroenteritis worldwide, and contaminated chicken is a significant vehicle for spread of the disease. This study aimed to assess consumers’ knowledge of safe chicken handling practices and whether their expectations for food safety labelling of chicken are met, as a strategy to prevent campylobacteriosis.

**Methods:**

We conducted a cross-sectional survey of 401 shoppers at supermarkets and butcheries in Wellington, New Zealand, and a systematic assessment of content and display features of chicken labels.

**Results:**

While 89% of participants bought, prepared or cooked chicken, only 15% knew that most (60–90%) fresh chicken in New Zealand is contaminated by *Campylobacter*. Safety and correct preparation information on chicken labels, was rated *‘very necessary’* or *‘essential’* by the majority of respondents. Supermarket chicken labels scored poorly for the quality of their food safety information with an average of 1.7/5 (95% CI, 1.4–2.1) for content and 1.8/5 (95% CI, 1.6–2.0) for display.

**Conclusions:**

Most consumers are unaware of the level of *Campylobacter* contamination on fresh chicken and there is a significant but unmet consumer demand for information on safe chicken preparation on labels. Labels on fresh chicken products are a potentially valuable but underused tool for campylobacteriosis prevention in New Zealand.

**Electronic supplementary material:**

The online version of this article (10.1186/s12889-018-5322-z) contains supplementary material, which is available to authorized users.

## Background

*Campylobacter* is a leading cause of human enteric infection, and its rising incidence in many parts of the world poses a significant public health concern [1]. It is the most common bacterial cause of diarrhoeal disease worldwide and the most common foodborne pathogen in many high income countries [2, 3]. In addition to gastroenteritis, potential sequelae of *Campylobacter* infection include hepatitis, pancreatitis, and Guillain-Barré syndrome [3]. In New Zealand, the burden of *Campylobacter* is significant, with 162 cases per 100,000 population in 2016 [4]. At least half of campylobacteriosis cases in New Zealand are attributable to contaminated chicken [5], and increasing levels of antimicrobial resistance in *Campylobacter* derived from chicken [6] raises concerns for future treatment of infection in humans.

Campylobacteriosis is at least partially preventable through improvements in consumer preparation of chicken products [7–9]. For example, Cogan et al. found that using hot water and detergent to clean hands and utensils after chicken preparation achieved a 50% reduction in *Campylobacter* contamination [9]. More broadly, greater awareness of food safety through the media has been shown to correlate with improvements in home food handling practices [10]. Therefore, ensuring consumers know correct techniques for safe chicken preparation is an important strategy in addressing high rates of campylobacteriosis.

Mandatory food safety labelling is a potential strategy to inform consumers of safe chicken preparation techniques. Labels have excellent consumer reach [11], unlike television, radio or internet advertisements which require device access, and are not widely used by some socio-demographic groups [12]. Labelling can be a cost-effective intervention [13, 14], as the cost can be borne by the producer provided they have a sufficiently long compliance period [15]. Moreover, government-mandated label content encourages food producer accountability [16] and is likely to be trusted by consumers [17], unlike food safety information from friends, relatives [12], retailers brochures or advertisements [17]. Providing information on contamination levels of retail chicken has been used in the United Kingdom as a strategy to increase accountability by chicken producers and retailers [18]. However, it is unknown whether current chicken labels meet the food safety needs and expectations of consumers. This is important because the attention given by consumers towards different forms of food safety information on labels (for example traceability barcodes versus certified quality marks) vary, and are not always predictable [19].

The aims of this study were to: 1) assess consumer knowledge of safe chicken preparation, 2) assess consumer expectations for food safety content on chicken labelling, and 3) investigate if these expectations are being met by current chicken labelling in New Zealand.

## Methods

### Study design

First, to assess consumer needs and expectations for information on safe chicken preparation on food labels, we conducted a street-intercept survey of supermarket and butchery shoppers. Second, to investigate whether these needs and expectations are being met, we developed a novel scoring system and applied this to analyse the quality of current raw chicken product labelling.

### Street-intercept survey of shoppers

To sample a population at risk of exposure to *Campylobacter*-contaminated chicken, we surveyed 401 grocery shoppers. Surveys were carried out from 19th–25th April 2016 at entrances to 12 supermarkets (comprising four major New Zealand supermarket chains) and six butcheries. Surveys were conducted throughout the day (supermarkets 0900–2100 h, and butchers 0900–1700 h) across the cities of Wellington, Lower Hutt and Porirua, New Zealand to encompass differing shopper demographics [20, 21]. All participants were at least 16 years old and provided written informed consent prior to completing our survey. Ethics approval was obtained from the Department of Public Health, University of Otago (reference D16/100)

Trained surveyors asked participants standardised questions on knowledge of safe chicken preparation by true/false statements, and views on content of chicken labelling using a 5-point Likert scale (*‘unnecessary’* to *‘essential’*). To isolate display features of labels [22], we standardised information content of three mock-up labels varied by design, and asked participants to select the most effective mock-up at communicating safe chicken preparation information to them. The mock-ups are presented in the supplementary materials (see Additional file 1), and comprised a typical current label, a current label with larger font, and a brightly-coloured warning label. The survey form is presented in the supplementary materials (see Additional file 2). Survey results are presented as percentages (responses/sample size) unless otherwise stated. Based on New Zealand census data, we estimated that a sample size of 384 survey participants would provide a 5% margin of error for the 50% figure at an alpha level of 0.05.

### Chicken product food safety labelling analysis

We assessed the quality of labelling of fresh chicken products available for purchase at the same locations at which street-intercept surveys were conducted. At each site, all labelled fresh chicken products were photographed for subsequent analysis. Duplicate labels, i.e. identical labels on different products of the same brand, were excluded. We also excluded frozen and cooked chicken products as these have significantly lower *Campylobacter* contamination levels [23].

We scored display and content features of labels, as they must be both legible and informative to be useful to consumers [24–26]. Display and content were each allocated up to five points. The display score gauged aspects such as label position, use of graphics and adequate font size of safety information. Adequate font size was given a higher maximum score as small text size is consistently identified as a barrier to label use [13]. For the content score, labels ranked higher if information such as minimum cooking duration or avoidance of chicken cross-contamination of other foods was present. The scoring system is detailed in the supplementary materials (see Additional file 3). Two assessors performed scoring, having each scored a test sample of 10 labels which confirmed sufficient interrater reliability. Content scores were plotted against display scores and the resulting graph was divided into poor, moderate and excellent segments by dividing each axis into equal thirds. For comparison with existing chicken labels, we also analysed the three mock-up labels used in our survey of shoppers.

## Results

### Street-intercept survey of shoppers

We approached a total of 584 shoppers, of whom 69% (401/584) agreed to be surveyed. Non-participants were not asked to provide a reason for refusal. 396 participants completed the survey, with minimal data missing due to participants refusing to provide a response, leaving prior to the end of the survey, or surveyor error. All available data for each question was analysed. The median age group of participants was 40–49 years (IQR 20–29 to 60–69 years) and 62% (246/396) of shoppers surveyed were female (Table 1). 82% (329/399) of participants were the main shopper for their household. The New Zealand Deprivation Index [21] was used to quantify socioeconomic status, with deciles 1–5 (less deprived) comprising 61% of participants (235/385) and deciles 6–10 (more deprived) comprising 39% (150/385).

**Table 1:** Demographic characteristics of survey participants

Age, years^a^	
16–19	20 (5%)
20–29	90 (22%)
30–39	60 (15%)
40–49	60 (15%)
50–59	66 (17%)
60–69	56 (14%)
70–79	34 (9%)
80+	11 (3%)
Sex^a^	
Female	246 (62%)
Refused	1 (< 1%)

^a^397 responses

Values are n (%)

The majority of participants (89%, 356/399) indicated that they bought, prepared or cooked chicken, and 83% (331/399) bought fresh raw chicken. Most responded correctly to questions about thorough cooking of raw chicken (99%, 393/397), preparing chicken using a separate knife and chopping board from other ingredients (97%, 386/398), cleaning and disinfecting the kitchen bench after being in contact with fresh raw chicken (95%, 379/398), and the use of hot tap water alone being insufficient for cleaning items after contact with fresh raw chicken (74%, 293/398). However, only 55% (219/398) of participants knew that *‘rinsing fresh raw chicken under the tap will reduce your likelihood of getting sick from it’,* was incorrect, while 23% (91/398) did not know. The most common response to *‘how much of the fresh raw chicken for sale in New Zealand do you believe has* Campylobacter *on it?’* was *‘some (10–40%)’* at 34% (133/392) while the correct answer of *‘most (60–90%)*’ [27] was selected by only 15% of participants (59/392). 23% (92/398) correctly stated that frozen chicken has less *Campylobacter* than fresh, but 43% (172/398) did not know.

**Fig. 1:**
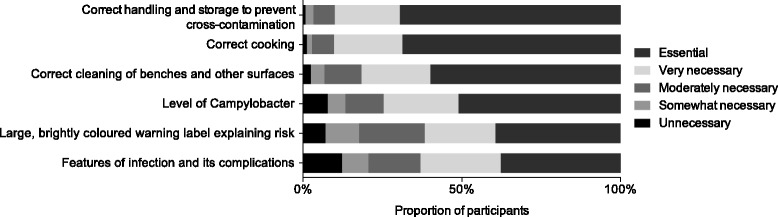
Participant views on the necessity of certain types of information on fresh chicken labels (397 responses)

Figure 1 presents the results for participants’ views on the necessity of different types of information on fresh chicken labels. All assessed information content was viewed by the majority of consumers as either *‘very necessary’* or ‘*essential’*. Specifically, 70% (276/397) of participants viewed information on the correct handling of chicken as *‘essential’* on labels, with a similar proportion reporting that instructions on correct cooking of chicken was also *‘essential’* (69%, 273/397). 60% (238/397) of participants believed information about cleaning of benches and other surfaces and correct cooking was *‘essential’* on labels, while 39% (156/396) considered it *‘essential’* that chicken products had *‘large, brightly-coloured warning labels to explain the risk of* Campylobacter*’*. Reporting the level of *Campylobacter* contamination was rated *‘essential’* by 51% (203/398), while 38% (150/397) considered stating features of *Campylobacter* infection and its complications on chicken labels to be ‘*essential*’.

When consumers were asked to select the test label (see Additional file 1) that most effectively communicated safe chicken preparation information to them, the majority picked the brightly-coloured label, label C (71%, 283/396). The ‘current’ chicken label, label A, was selected least often (< 1%, 2/396).

### Chicken-product food safety labelling analysis

We analysed 45 chicken labels for the quality of their food safety information content and presentation. Each supermarket chain tended to have similar labels for their range of on-site packaged raw chicken, but there was considerable variation between chains and for non-supermarket branded chicken. Labels contained information on weight, price, packaged-on date and use-by date, however information on safe handling of raw chicken varied from non-existent to moderate. Butchery products in general were labelled at time of purchase with a sticker identifying product type, weight and price but had no information on safe chicken handling.

**Fig. 2:**
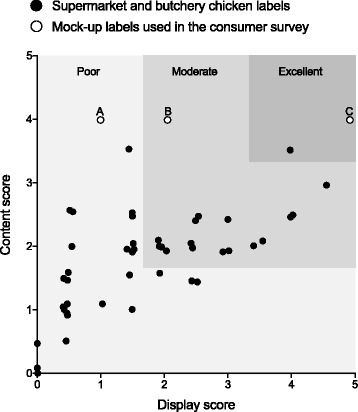
Display and content quality scores of current chicken labels, and consumer survey mock-up labels. Plot of content score against display score for all analysed supermarket and butchery chicken products, and mock-up labels used in the consumer survey. Mock-up labels are marked, (A) current label, (B) current label with larger text size, and (C) brightly-coloured warning label. For the purposes of display, data points have been jittered to reveal overlapping points

Supermarket raw chicken labels had an average content score of 1.7 out of 5 (95% CI, 1.4–2.1) and display score of 1.8 out of 5 (95% CI, 1.6–2.0). Butchery raw chicken labels all scored 0 out of 5 for content and 0 out of 5 for display. Figure 2 shows how labels were distributed according to their quality. Almost all fell within the poor to moderate range. Label mock-ups all scored 4 out of 5 for content. For display features, mock-up label A scored 1 out of 5, label B scored 2 out of 5 and label C scored 5 out of 5.

## Discussion

This study assessed consumer perspectives on chicken labelling with a concurrent analysis of existing labels at outlets at which survey participants were shopping. This meant that consumer views could be compared with the quality of chicken labels to which the consumers were exposed. Our response rate of 69% is high for street-intercept surveys. The median age group in our sample was 40–49 years and 62% of shoppers surveyed were female, in line with the typical New Zealand household shopper (Nielsen Consumer and Media Insights, personal communications, April 2016). The results of this study are likely to be applicable to other high-income countries where contaminated chicken products are a major source of *Campylobacter* infection.

We found that, while food safety knowledge was generally good, many consumers have important gaps in their knowledge of chicken product safety. Only 55% of respondents were aware that rinsing fresh raw chicken under the tap does not reduce the likelihood of illness, and only 15% knew that 60–90% [27] of retail chicken meat in New Zealand is contaminated with *Campylobacter*. Previous studies have also identified areas for improvement of consumers’ knowledge of food safety. A recent literature review of food safety knowledge and behaviour of Canadian consumers reported good knowledge about safe meat preparation, but also identified specific opportunities to improve handling practices, for example the use of a thermometer to check internal meat temperature [28].

Our findings demonstrate that current supermarket and butchery chicken labels have poor safety information content. Consumers expressed a desire for specific food safety information such as the level of *Campylobacte*r contamination on chicken. Consumers also want this information displayed effectively, and identified a mock-up label with prominently displayed safety information as the most effective at conveying food safety advice, in comparison to current labels. In our label analysis, we found that this information was entirely absent on products from some retailers, or if present was often difficult to read. Similar variability in label content and display quality has been identified previously. An analysis of food labelling in Canada reported that improving the consistency of food label safety information display, such as typography and location on the packaging, would improve consumer access to this information [25]. The study also recommended stricter regulations regarding legibility [25].

Our results demonstrate that consumer demand for safety information on chicken products is not being met by current chicken labelling. This deficiency in safety information may relate to the fact that New Zealand and Australian labelling standards do not mandate provision of preparation or storage information on chicken products, instead advocating “promotion of food safety” to prevent illness from *Campylobacter* [29]. Similar regulatory gaps exist in the United Kingdom and Canada where chicken handling information is also not mandated on labels [30, 31]. This deficiency suggests that market self-regulation of labelling is insufficient to meet consumer needs and expectations. Consequently, policy changes appear necessary, including mandatory disclosure of *Campylobacter* risk, to ensure industry accountability for chicken quality and to meet consumer demand for safety information.

The effect of brightly-coloured, informative warning labels on chicken products is likely to be two-fold. As well as informing consumers of *Campylobacter* risk and prevention measures, labelling of chicken products, particularly if mandatory, is likely to incentivise industry measures to reduce *Campylobacter* levels. In the United Kingdom, Public Health England identified the lack of pressure on the chicken industry as a barrier to *Campylobacter* reduction and consequently recommended mandating disclosure of *Campylobacter* levels to consumers [18]. Introduction of this strategy saw an overall decline in the amount of chicken contaminated with the highest levels of *Campylobacter* from 20% in 2014 to 7% in 2017, resulting in an estimated 100,000 fewer campylobacteriosis cases per year [32]. Implementation of a mandatory labelling scheme should be supported by other initiatives such as education campaigns. The impact and value of these interventions should be evaluated with qualitative and quantitative research to assess whether consumers understand what they are reading on labels, whether it changes their behaviour, and whether it impacts disease rates. An economic evaluation of the labelling change along with other interventions would also be useful to better understand the benefits of these approaches, which can be very large compared with their costs [33]. Ultimately it may be more effective to directly regulate and enforce production standards to lower contamination levels in fresh chicken. Such interventions have been shown to be highly effective (halving the rate of campylobacteriosis in New Zealand during 2007 within months of being implemented) [5] and almost certainly have the greatest potential to reduce disease burden if fully implemented.

The results of this study should be interpreted in the context of some methodological considerations. First, we cannot exclude the possibility that social desirability bias may have influenced participants to identify an aspect of chicken label information as *‘essential’*, believing that this would be viewed favourably by the surveyor. However, given the topic of the survey was non-personal, the impact of social desirability bias is likely to be minimal. Second, providing information regarding the extent of *Campylobacter* contamination of chicken may have influenced participants to express a desire for more informative and eye-catching labels. However, in designing this study it was hypothesised that many respondents would be unaware of *Campylobacter* levels and would require this information to respond to the survey. Third, we asked consumers which mock up label was ‘most effective’ for them personally. It is unclear if consumers interpreted this to mean their preferred label or the more ‘attention grabbing’ display, which was evidently label C (see Additional file 1). Nonetheless, attracting consumer attention is essential for delivering warning messages, and bright colours are known to be effective at achieving this purpose [34].

## Conclusions

Campylobacteriosis from contaminated chicken meat is one of the most important food safety problems in western countries, and dissemination of antibiotic resistant organisms is a growing concern. It is also a preventable disease. Food labels are a universally accessible means of conveying safe chicken preparation information to consumers. Our research identified demand for comprehensive safe chicken preparation and handling information on labels and demonstrated several gaps in consumer knowledge. Consumers currently underestimate the level of *Campylobacter* contamination on fresh raw chicken, and have stated a desire to have such information presented on labels to inform their purchasing decisions. Furthermore, our chicken label analysis demonstrated a lack of consistent safety messages in an easily-useable format, highlighting a key deficiency to be addressed. We recommend mandatory introduction of comprehensive, high-quality, chicken safety labelling, along with evaluation to establish whether this intervention leads to changes in consumer behaviour and reductions in the incidence of *Campylobacter* infection.

## Additional files

Additional file 1: Chicken mock-up labels presented to consumers. (A) Current standard chicken label. (B) Current standard chicken label with information displayed in a larger font size. (C) Brightly-coloured warning label with large bold font, separate from the price and/or weight label. Consumers were asked to state which mock-up label was most effective at presenting information on safe chicken preparation to them. Note that information content was standardised across all three mock-up labels in order to isolate display differences. (EPS 1367 kb)

Additional file 2: Survey form. (DOCX 85 kb)

Additional file 3: Chicken label analysis criteria. (DOCX 19 kb)
