# Supplementary material for: Food safety labelling of chicken to prevent campylobacteriosis: consumer expectations and current practices
Source: BMC Public Health. 2018 Mar 27;18:414. doi: 10.1186/s12889-018-5322-z (PMC5870189; doi:10.1186/s12889-018-5322-z)
Supplement: Supplementary file 2 — Survey form. (DOCX 85 kb) [file 12889_2018_5322_MOESM2_ESM.docx]

**Additional file 2: Survey form**

**SURVEY FORM**

**A. Interview details**

**Informed consent gained: Yes** ☐

1. Interviewer
2. Location
3. Date Time
4. Are you the main shopper for your household? Yes ☐ No ☐

**B. Categories of information on food labels**

*Read: Food labels can have a variety of information displayed on them. Please look at this show card and tell me how necessary you think it is that labels have the following information:*

***[SHOWCARD 1]****(Necessary statements. NB. if necessary remind them that we are referring to label information)*

1. Food safety to protect from infectious diseases, e.g. proper handling and storage

1 ☐ 2 ☐ 3 ☐ 4 ☐ 5 ☐

1. Safety for vulnerable groups, e.g. people with nut allergies

1 ☐ 2 ☐ 3 ☐ 4 ☐ 5 ☐

1. Nutritional composition, e.g. percentage of sugar, salt and saturated fat

1 ☐ 2 ☐ 3 ☐ 4 ☐ 5 ☐

1. Energy in food, e.g. distance needed to walk to ‘burn off’ calories per serving

1 ☐ 2 ☐ 3 ☐ 4 ☐ 5 ☐

1. Additives, e.g. preservatives, colouring agents

1 ☐ 2 ☐ 3 ☐ 4 ☐ 5 ☐

1. Environmental impact and sustainability, e.g. greenhouse gas (carbon) footprint

1 ☐ 2 ☐ 3 ☐ 4 ☐ 5 ☐

1. Animal welfare, e.g. free range

1 ☐ 2 ☐ 3 ☐ 4 ☐ 5 ☐

1. Welfare of workers involved in production, e.g. fair trade

1 ☐ 2 ☐ 3 ☐ 4 ☐ 5 ☐

1. Information for those with specific dietary preferences & religious beliefs, e.g. vegetarian, halal

1 ☐ 2 ☐ 3 ☐ 4 ☐ 5 ☐

1. Value for money, e.g. cost per 100g or cost per kg

1 ☐ 2 ☐ 3 ☐ 4 ☐ 5 ☐

1. Country of origin labelling to help consider food safety risks and other concerns eg, animal welfare, welfare of workers

1 ☐ 2 ☐ 3 ☐ 4 ☐ 5 ☐

**C. General views on food labels**

*Read: I’d now like to ask you about your general view on food labels. Please look at this show card and indicate your level of agreement with the following statements:*

***[SHOWCARD 2]****(Agreement statements)*

1. Excluding the price label, I always read food labels before buying a food product for the first time

1 ☐ 2 ☐ 3 ☐ 4 ☐ 5 ☐

1. I believe food labels currently contain all the information I need

1 ☐ 2 ☐ 3 ☐ 4 ☐ 5 ☐

1. I believe the government should prohibit certain foods from being in the market if they are, for example, unhealthy for people or bad for the environment

1 ☐ 2 ☐ 3 ☐ 4 ☐ 5 ☐

1. I believe the government should tax unhealthy foods and drinks, e.g. sugar sweetened beverages

1 ☐ 2 ☐ 3 ☐ 4 ☐ 5 ☐

1. I believe consumers should be given all the information about foods so that they can decide themselves, e.g. each product has a link to a website with this information

1 ☐ 2 ☐ 3 ☐ 4 ☐ 5 ☐

**D. Knowledge of fresh chicken**

*Read: We now have some more specific questions to ask you about one type of food, chicken.*

1. Do you buy, prepare or cook chicken? Yes ☐ No ☐
2. If yes, which of the following chicken products do you buy, prepare or cook?
   1. Fresh raw chicken Yes ☐ No ☐
   2. Frozen raw chicken Yes ☐ No ☐
   3. Cooked chicken Yes ☐ No ☐
   4. Other chicken products (specify) Yes ☐ No ☐
3. How often do you typically prepare fresh raw chicken at home?
   1. Daily ☐
   2. 2-4 times per week ☐
   3. Once per week ☐
   4. 1 to 3 times per month ☐
   5. Less than once per month ☐
   6. Never ☐
4. Did you know that fresh raw chicken can carry bacteria (bugs) that can make you sick? Yes ☐ No ☐
   1. If yes, can you tell me the name of the bacteria (bug)? Specify:
   2. If no, or named different bacteria, have you heard about c………r? Yes ☐ No ☐
5. How much of the fresh raw chicken for sale in New Zealand do you believe has c………r on it? *(NB. Best guess)*
   1. Little or none (<10%) ☐
   2. Some (10-40%) ☐
   3. About half (40-60%) ☐
   4. Most (60-90%) ☐
   5. Almost all or all (>90%) ☐
6. For the following statements about safe chicken preparation please tell me which ones you think are true or false, or are ones you ‘don’t know’?
   1. You should use a separate knife and chopping board for fresh raw chicken from other ingredients

T ☐ F ☐ Don’t know ☐

- 1. Hot tap water is sufficient to clean anything that comes into contact with fresh raw chicken

T ☐ F ☐ Don’t know ☐

- 1. Fresh raw chicken must be cooked through before eating

T ☐ F ☐ Don’t know ☐

- 1. Rinsing fresh raw chicken under the tap will reduce your likelihood of getting sick from it

T ☐ F ☐ Don’t know ☐

- 1. You should clean and disinfect your kitchen bench after contact with fresh raw chicken

T ☐ F ☐ Don’t know ☐

- 1. Frozen raw chicken has less campylobacter than fresh raw chicken

T ☐ F ☐ Don’t know ☐

**E. Views on content of labelling of fresh chicken**

*Read: I now want to ask you about your views on labelling of fresh chicken. Just to let you know, most fresh raw chicken products sold in New Zealand are contaminated with campylobacter. Please look at this show card and tell me how necessary you think it is that labels on fresh chicken have the following information features:*

***[SHOWCARD 1]****(Necessary statements. NB. If asked, “most” is 60-90%)*

1. The level of campylobacter on that particular product

1 ☐ 2 ☐ 3 ☐ 4 ☐ 5 ☐

1. Features of campylobacter infection and its complications

1 ☐ 2 ☐ 3 ☐ 4 ☐ 5 ☐

1. Correct cooking to kill campylobacter

1 ☐ 2 ☐ 3 ☐ 4 ☐ 5 ☐

1. Correct handling and storage to prevent campylobacter cross-contamination of other foods

1 ☐ 2 ☐ 3 ☐ 4 ☐ 5 ☐

1. Correct cleaning to kill campylobacter on benches and other surfaces

1 ☐ 2 ☐ 3 ☐ 4 ☐ 5 ☐

1. Large, brightly coloured warning labels on chicken products to explain the risk of campylobacter

1 ☐ 2 ☐ 3 ☐ 4 ☐ 5 ☐

**F. Fresh chicken label mock-ups**

*Read: We have a current typical label and prepared sample mock-ups of possible fresh raw chicken labels to show you. Please look at this show card and tell me which you think is most effective at communicating safe chicken preparation information to you:*

***[SHOWCARD 3]****(Mock-ups. NB. Read out options on showcard)*

A. Current label ☐

B. Current with extra information ☐

C. Separate ‘pronounced’ label ☐

**G. Participant details**

*Read: We just need a few details about you:*

1. Age group: 16-19 20-29 30-39 40-49 50-59 60-69 70-79 80+
2. Sex: M ☐ F ☐
3. Which suburb do you live in?:
4. Which ethnic group do you belong to? Indicate the group or groups which apply to you ***[SHOWCARD 4]****(Ethnicity. NB. Multiple responses are possible)*

a. New Zealand European b. Māori

c. Samoan d. Cook Island Māori

e. Tongan f. Niuean

g. Chinese h. Indian

i. Other such as DUTCH, JAPANESE, TOKELAUAN. Please state:

j. Don’t know k. Refused

*Read: That is the end of the survey. Do you have any comments or questions?*

Notes:

**Survey Ends**
