# Supplementary material for: Food safety labelling of chicken to prevent campylobacteriosis: consumer expectations and current practices
Source: BMC Public Health. 2018 Mar 27;18:414. doi: 10.1186/s12889-018-5322-z (PMC5870189; doi:10.1186/s12889-018-5322-z)
Supplement: Supplementary file 3 — Chicken label analysis criteria. (DOCX 19 kb) [file 12889_2018_5322_MOESM3_ESM.docx]

**Additional file 3: Chicken label analysis criteria**

Collected photographs of labels were scored by **display** and **content**, each of which received up to 5 points, for a total score out of 10.

First, for the **display score**, points were allocated as follows (score):

1. Demarcation of safety information from other text (0.5)

2. Positioning the label on the front side of the product package (0.5)

3. The use of a contrasting colour surrounding the safety information (0.5)

4. The use of graphics relating to safe handling of raw chicken (0.5)

5. The use of simple language (i.e. no jargon or potential for misinterpretation) (0.5)

6. Clear structure and layout of safety information (0.5)

7. Font size of the safety information wording based on absolute size and size relative to that of other label information. Font size was scored as 0.5, 1.0 or 2.0. This expanded range of scores is in cognisance of small font sizes being consistently identified by consumers as a barrier to label use [1].

a. Small font size (~3 mm in height). This is the minimum size permissible by Food Standards Australia New Zealand (0.5)

b. Moderate font size (>3 mm in height but smaller than other wording on the label) (1.0)

c. Large font size (>3 mm in height *and* equivalent or larger in size than other wording on the label) (2.0)

Second, for the **content score**, we used information elements from the ‘Cook, Clean, Cover and Chill’ strategy supported by the Ministry for Primary Industries, the governmental department responsible for food safety enforcement in New Zealand. We also incorporated extra information deemed important in the safe handling of raw poultry [2]. Content scores were allocated as follows (score):

1. The ‘Cook’ category

a. Mentioning the need to cook the product before consumption (0.5)

b. Detailing a set duration and temperature for suitable cooking, or the recommendation that juices from the chicken should run clear prior to consumption (0.5)

2. The ‘Clean’ category

a. Mentioning that items or surfaces that come in contact with raw poultry must be cleaned (0.5)

b. Details on how to appropriately clean items or surfaces to ensure *Campylobacter* is killed (0.5)

3. The ‘Cover’ category

a. Mention of ensuring the raw product does not come into contact with other foods (0.5)

b. Mentioning the need to cover raw product or place in a container for storage (0.5)

4. The ‘Chill’ category

a. Mentioning the need to refrigerate raw product (0.5)

b. Specification of a range of suitable temperatures for refrigeration (0.5)

5. Extra information regarding the safe handling of raw chicken identified as important based on our review of the literature [3]

a. Description of appropriate freezing and thawing instructions (0.5)

b. Advice against the rinsing of the raw product under the tap, a common behaviour that aids the spread of *Campylobacter* across kitchens [2] (0.5)

**References**

1. Campos S, Doxey J, Hammond D. Nutrition labels on pre-packaged foods: a systematic review. Public health nutrition. 2011;14(8):1496-1506.

2. Henley SC, Gleason J, Quinlan JJ. Don't wash your chicken!: A food safety education campaign to address a common food mishandling practice. Food Protection Trends. 2016;36(1):43-53.

3. Ministry for Primary Industries. Food safety in the Home. Wellington: Ministry for Primary Industries; 2012.
